# Management of Enamel Defects with Resin Infiltration Techniques: Two Years Follow Up Retrospective Study

**DOI:** 10.3390/children9091365

**Published:** 2022-09-08

**Authors:** Alessia Vincenza Brescia, Lorenzo Montesani, Dimitri Fusaroli, Raffaella Docimo, Gianfranco Di Gennaro

**Affiliations:** 1 Paediatric Dentistry, Doctoral School in Materials for Health, Environment and Energy, University of Rome “Tor Vergata”, 00133 Rome, Italy; 2 Independent Researcher, 00187 Rome, Italy; 3 Department of Systems Medicine, University of Rome “Tor Vergata”, 00133 Rome, Italy; 4 Paediatric Dentistry, Department of Surgical Sciences, University of Rome “Tor Vergata”, 00133 Rome, Italy; 5 Department of Health Sciences, University of Magna Graecia, 88100 Catanzaro, Italy

**Keywords:** paediatric dentistry, resin infiltration, dental enamel, visual assessment

## Abstract

Background: Developmental Defects of Enamel (DDE) represent an aesthetic, functional, and often psychological problem, especially in young patients. Infiltrative treatment with resin (Icon-DMG, Hamburg, Germany) is a minimally invasive technique based on the infiltration of high viscosity resin inside the hypomineralized enamel, modifying its optical properties. The aim of this paper is to evaluate the clinical efficacy of superficial infiltration in the treatment of white enamel defects of the anterior sector with pre-eruptive etiology and its stability over time. Methods: Thirty-three patients affected by DDE associated with mild and moderate Molar Incisor Hypomineralization (MIH), mild and moderate fluorosis, and post-traumatic hypomineralization treated with resin infiltration were retrospectively retrieved. Results: In all cases an improvement in aesthetic appearance was achieved, and the 24-month follow-up confirmed the stability of the results. However, in the cases of traumatic hypomineralization the results were not completely satisfactory. Conclusions: The superficial infiltration technique can be considered a valid minimally invasive alternative to traditional treatment of mild or moderate fluorosis and mild MIH.

## 1. Introduction

Developmental Defects of Enamel (DDE) represent an aesthetic, functional, and often psychological problem, especially in young patients. In a study of adolescents, it was found that they frequently hide their smiles or even limit their social life due to opacity of the enamel of the frontal sector [1]. DDE can alter the aesthetics of the smile, sensitivity, and occlusal function, representing a risk factor for the development of tooth decay and erosions [2]. DDE can be histologically divided into the two categories of quantitative defects and qualitative defects [2,3]. The former is represented by enamel hypoplasia or incomplete enamel formation due to alteration of the adamantoblasts, which is associated with a reduction of enamel thickness and its resistance [4]. However, hypomineralization of the enamel is a qualitative defect that appears as an abnormal translucency or opacity visibly present on the crown of the affected teeth. From an etiological point of view, enamel defects can be divided into pre-eruptive and post-eruptive defects [1,5].

The pre-eruptive enamel defects included in this study are associated with Molar-Incisor Hypomineralization (MIH), Fluorosis, and post-traumatic hypomineralization. Pre-eruptive defects have a prevalence ranging from 9% to 68%, and represent the consequence of abnormal function of ameloblasts during the amelogenesis process [3,6]. The objectives of treatment consist in the resolution of the symptoms and the aesthetic, morphological, and functional restoration of the affected teeth by means of a multidisciplinary approach [7]. The infiltrative treatment with resin (Icon-DMG, Hamburg, Germany) is a minimally invasive technique that is not based on the removal of the dysplastic enamel; instead, it uses infiltration of high viscosity resin inside the hypomineralized enamel, modifying its optical properties [8]. The infiltration of the resin increases the refractive index of the white lesions, which consequently take on the appearance of the surrounding healthy enamel, restoring its natural translucency [9,10]. The aim of the present study was to evaluate the clinical efficacy of superficial infiltration with ICON (DMG, Hamburg, Germany) in the treatment of white enamel defects of the anterior sector and with pre-eruptive etiology associated with mild and moderate degree of MIH, a mild and moderate degree of fluorosis, or post-traumatic hypomineralization, analyzing:

- Aesthetic results and his stability over time;
- Reduction of hypersensitivity, if present.

## 2. Materials and Methods

### 2.1. Sample Description

Thirty-three patients (18 M; 15 F) affected by white enamel defects of pre-eruptive etiology associated with mild and moderate MIH, mild and moderate fluorosis, and post-traumatic hypomineralization and who had been treated with resin infiltration “ICON (DMG, Hamburg, Germany)” between January 2020 and July 2020 were retrospectively retrieved from our database.

To evaluate the effectiveness of the infiltrating resin treatment in terms of both aesthetics and sensitivity, only defects related to the anterior sector were considered.

The inclusion criteria were as follows:

- Patients subjected to infiltrative treatment with resin (ICON-DMG) from January 2020 to July 2020.
- Elements of anterior sector affected by mild or moderate degree of MIH (according to the classification of Mathu-Muju and Wright) [11].
- Elements of anterior sector with mild or moderate degree of fluorosis (according to the Thylstrup and Ferjerskov index) [12].
- Elements of anterior sector with post-traumatic hypomineralization.

The exclusion criteria were:

- Elements with conservative treatments;
- Cavitated enamel defects;
- Enamel defects with post-eruptive etiology;
- Severe degree of MIH;
- Severe degree of Fluorosis.

### 2.2. Infiltrative Resin Treatment Procedure

At T0 (from January 2020 to July 2020), the dental elements were treated with ICON Kit Vestibular (DMG, Hamburg, Germany) according to the following procedure.

Teeth were cleaned with a prophy cup and isolated by a rubber dam, and the resin infiltration technique was performed according to the manufacturer’s indications: treated opacities were etched for 2 min with 15% hydrochloric acid (Icon Etch, DMG, Hamburg, Germany) and rinsed with air–water spray for 30 s. Desiccation of the lesions was performed by air blowing for 10 s followed by application of ethanol 99% (Icon Dry, DMG, Hamburg, Germany) for 30 s on the conditioned surface. This allowed the removal of water particles present in the pores of the enamel and pre-visualization of the result, while ethanol has a refractive index similar to that of the resin applied subsequently. It was necessary to repeat the etching and ethanol application step three consecutive times. Subsequently, infiltrating resin was applied for 3 min (Icon-Infiltrant, DMG, Hamburg, Germany). The excess resin was removed with air-spray and dental floss. Finally, photopolymerization was performed for 40 s. The resin infiltration phase was repeated a second time with an infiltration time of 60 s to allow the resin to penetrate the residual porosities. Finally, photopolymerization was performed for a further 40 s and polishing was performed with silicone discs and rubber pads. Follow-up sessions were scheduled at 12 and 24 months to verify that the stability of the aesthetic result obtained a T1 as well as the degree of hypersensitivity of the treated dental elements.

At T1 (after 1 year) a follow-up was performed to assess the stability of the result obtained at T0 and to verify any reduction in hypersensitivity.

At T2 (after 2 years) the sample selected according to the eligibility criteria was re-evaluated by follow-up. Three observers evaluated the aesthetic appearance of the teeth at T0, T1, and T2, using FDI color match criteria to standardize the diagnostic–therapeutic procedures.

### 2.3. Photographic Documentation and Chromatic Evaluation

Qualitative assessments were performed using digital photographs (Nikon D80, Nikon AF-S Micro Nikkor 105 mm 1:2:8 G lens and Nikon Wireless Remote SB-R200 speedlight flash) pre- and post-treatment with standard camera settings (speed shutter speed 1/50) and aperture settings (f32). In addition, the same lighting and imaging process were used. Three observers evaluated the aesthetic appearance of the teeth before and after treatment using FDI color match criteria. The color variation was obtained by calculating the difference in the score at T0, T1, and T2 of the color matching criteria approved by the “Fédération Dentaire Internationale” (FDI). The scale consists of five quality scores:

1: Clinically excellent/very good (Good color match. No difference in shade and translucency);
2: Clinically good (minor deviations);
3: Clinically sufficient/satisfactory (Clear deviation but acceptable. Does not affect aesthetics);
4: Clinically unsatisfactory (Localized-clinically unsatisfactory but can be corrected by repair);
5: Clinically poor (Unacceptable. Replacement necessary) [13].

### 2.4. Dentinal Hypersensitivity

Regarding pre- and post-treatment hypersensitivity, the following outcomes were collected:

- The Shiff Air Index was used to evaluate the perception of discomfort after the application of air using an air-spray syringe for 3 s at 2 mm of distance and perpendicular to the tooth surface.
- The Wong–Baker Faces Pain Rating Scale was used to characterize pain; this scale uses values ranging from 0 to 10, where 0 represents “no pain” and 10 represents “very strong pain”.

### 2.5. Statistical Analysis

Data were summarized by mean and standard deviation if normally distributed. Median and interquartile range were used in case of skewed distributions. The Shapiro–Wilk test was used to assess normality assumptions. Categorical variables were expressed as counts and percentages, and 95% confidence intervals of opacity prevalence were computed using the Clopper–Pearson method. Opacities were divided into three groups: mild or moderate MIH, mild or moderate fluorosis, and traumatic hypomineralization. We performed a repeated (three timepoints) restricted maximum likelihood mixed model regression on three outcomes: the Shiff Air Index, the Wong–Baker Faces Pain Rating, and FDI, using enamel opacity as a confounder. Levels of the marginal outcomes were estimated at each type of opacity and reported by graphs. No missing data imputation strategy was preplanned. Statistical significance was set at 5%. Analyses were conducted using Stata statistical software (16.1, StataCorp LLC, College Station, TX, USA).

## 3. Results

### 3.1. Study Population

According to the eligibility criteria, thirty-three patients (mean age 11 years) were retrieved from the institutional database. One hundred and fourteen dental elements were treated with the superficial infiltration technique using the Vestibular ICON Starter Kit (DMG, Hamburg, Germany). The characteristics of the sample are shown in Table 1.

### 3.2. Qualitative Visual Evaluation

**Figure 1:**
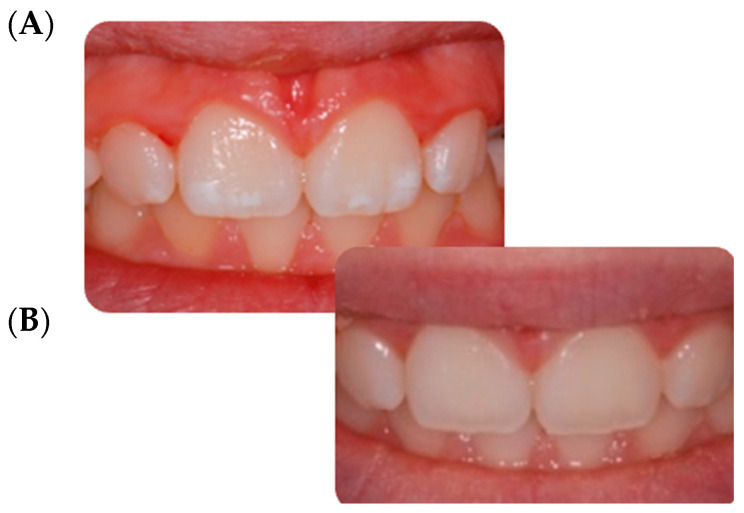
Mild MIH. (**A**) Opacities located on the central incisors’ buccal surfaces. (**B**) 24-month follow up.

**Figure 2:**
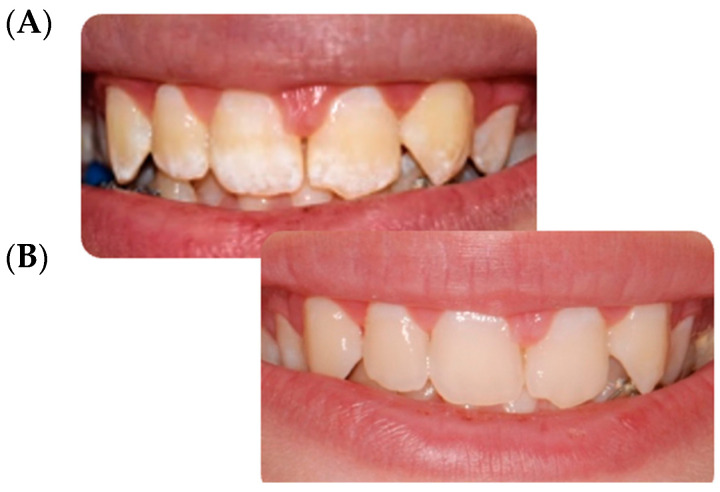
Fluorosis. (**A**) Opacities on all upper teeth especially located on the incisal and cervical tooth’s buccal surfaces. (**B**) 24-month follow up.

**Figure 3:**
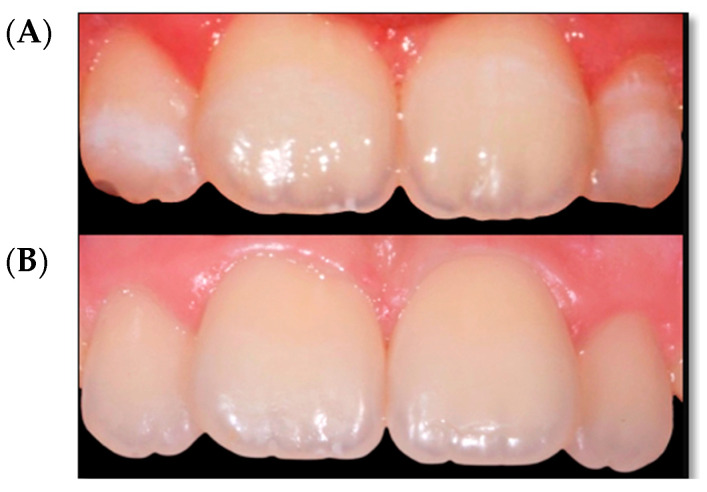
Mild MIH. (**A**) Opacities located on the lateral incisors’ buccal surfaces. (**B**) 24-month follow up.

**Figure 4:**
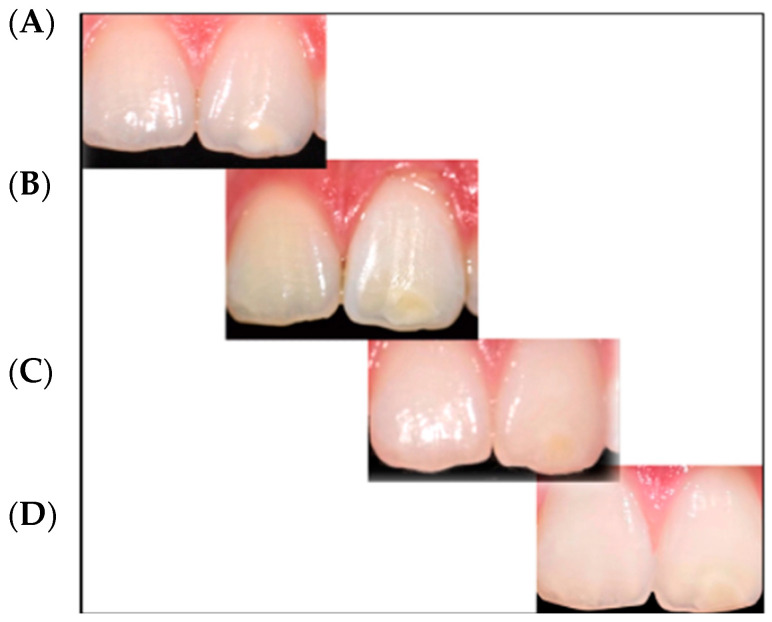
Post-trauma hypomineralization. (**A**) Opacities located on the left central incisor buccal surface. (**B**) Immediately after resin infiltration. (**C**) 12-month follow up. (**D**) 24-month follow up.

According to FDI color match criteria [13], the pre-treatment quality assessment was found to be “clinically unsatisfactory” (mean FDI 4.18). On the contrary, after the infiltration technique, the same evaluation was found to be “clinically excellent” (mean FDI 1.72). In all cases, an improvement in the aesthetic appearance was achieved (Figure 1, Figure 2, Figure 3 and Figure 4). Specifically, a “clinically excellent” result was found in 60.6% of cases, “clinically good” in 18.2%, “clinically sufficient” in 6.1%, and “clinically unsatisfactory” in 15.1% of cases. The 24-month follow-up confirmed the stability of the aesthetic results obtained over time, noting an improvement in several cases. The distribution of the pre- and post-treatment FDI scores among the whole sample is described in Table 2. Furthermore, considering the higher prevalence of MIH cases within the observation sample, it was possible to calculate the success rate of the infiltrative treatment within the group of patients affected by MIH, finding a “clinically excellent” result equal to 66.67%, “clinically good” equal to 20.83%, “clinically sufficient” equal to 8.3%, and “clinically insufficient” in 4.2% of cases.

Finally, all patients were satisfied, because although in certain cases the result obtained was not considered optimal, the result was nonetheless considered satisfactory by each patient thanks to both the attenuation of opacities and the improvement of dentinal hypersensitivity.

### 3.3. Dentin Hypersensitivity Assessment

At T0, 24.3% of patients had a value of the Wong–Baker Faces Pain Rating Scale between 6 and 10, indicating a discomfort from “strong” to “very strong”. After treatment (T1) a value less than 2 was found in 90.9% of patients, while after 24 months (T2) all patients showed a value equal to or less than 2 (Table 2). A significant improvement in dentinal hypersensitivity of the dental elements was found in all patients, especially those affected by MIH. For this reason, the result was considered satisfactory by each patient both for the attenuation of opacities and for the improvement of dentinal hypersensitivity.

### 3.4. Statistical Analysis

**Figure 5:**
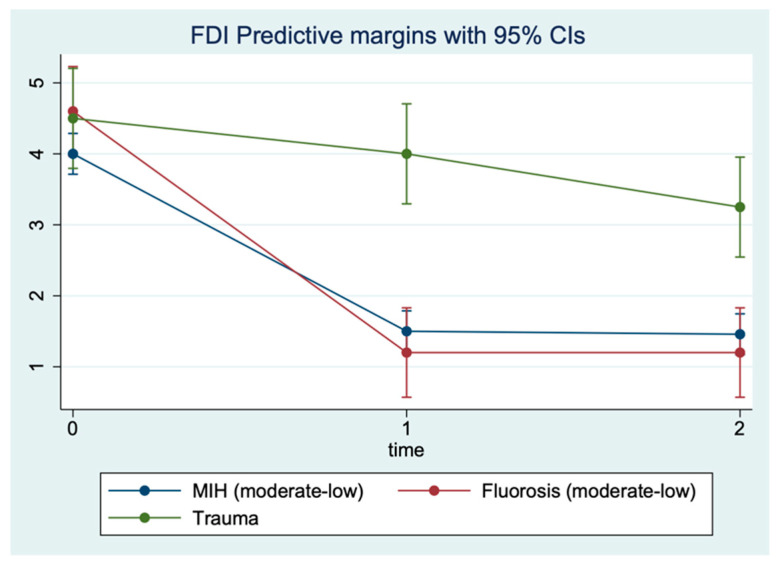
FDI levels predicted by repeated measures (three timepoints) restricted maximum likelihood mixed model.

As shown by the regression analysis (Figure 5), the aesthetic improvement at T1 and the stability of the results at T2 is significantly greater in patients with MIH and fluorosis than in patients with trauma. More precisely, the FDI value predicted by the regression model for patients with MIH decreases from 4.0 (T0) to 1.5 (T1) and remains almost constant at T2 (1.46). Similarly, for patients with fluorosis, the FDI value decreases from 4.6 (T0) to 1.2 (T1), which remains the same at T2. Conversely, in patients with trauma, FDI falls much less markedly, from 4.5 (T0) to 4 (T1) and to 3.25 (T2). This difference in trajectory between the MIH and fluorosis groups compared to trauma patients is statistically significant (*p* value of the interaction terms < 0.05, Table 3). Similarly, the trajectory of the Wong Scale shows statistically significant overall improvement of all patients at T1 and T2 compared to T0, with a decrease of 2.4 and 2.8 points, respectively (*p* < 0.001, Table 4). Unlike FDI, the trend does not change significantly between the three groups of patients (interaction terms *p* > 0.05), with all three groups reaching Wong values close to zero at T1 and T2.

**Figure 6:**
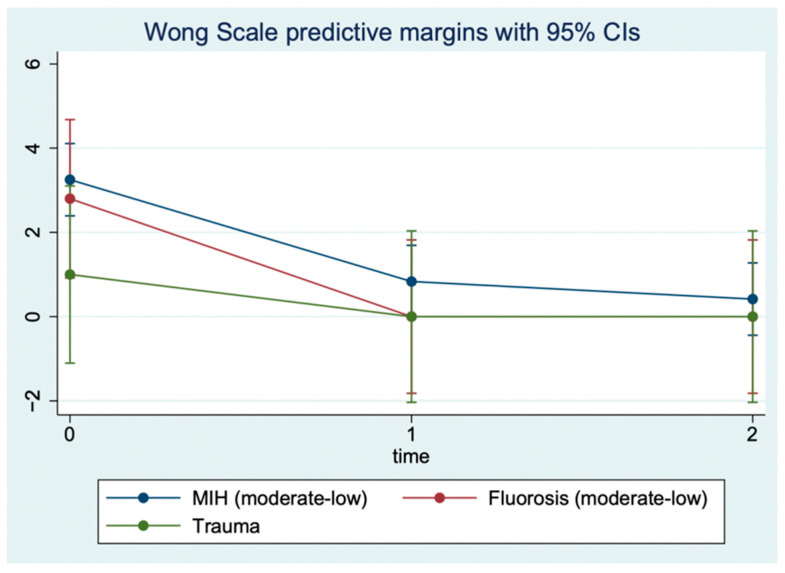
Wong–Baker Faces Pain Rating Scale levels predicted by repeated measures (three timepoints) restricted maximum likelihood mixed model.

**Figure 7:**
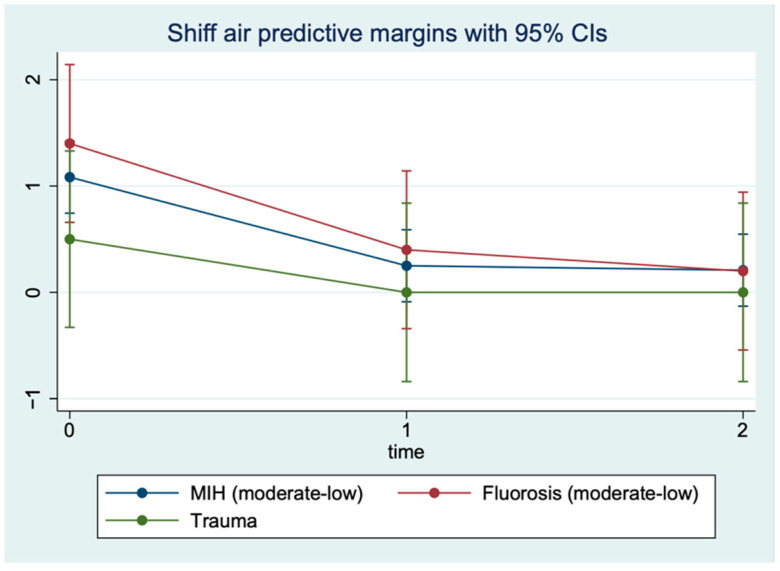
Shiff air index levels predicted by repeated measures (three timepoints) restricted maximum likelihood mixed model.

However, it should be noted that patients with trauma show a lower Wong score at baseline (T0) than patients with fluorosis and with MIH (Figure 6). Finally, for the Shiff-air values a trend emerges that is identical to that of the Wong scale values. The temporal trend is not significantly different between the three groups (interaction terms *p* > 0.05, Table 5), with values at T0 of 1.08 (MIH), 1.4 (Fluorosis), and 0.5 (Trauma) and at T1 of 0.25 (MIH), 0.4 (Fluorosis), and 0 (Trauma), remaining almost constant at T2 (MIH: 0.21; Fluorosis: 0.20; Trauma: 0.0) (Figure 7).

## 4. Discussion

The opacity of the enamel, regardless of etiological origin [14], differs from healthy enamel based on the refractive indices (RI) between the enamel crystals and the medium within the porosity that causes the dispersion of light, thus making evident opacities [3]. The aesthetic result of the infiltrative technique is based on the modification of the interaction of light with the enamel, and thereby the visual perception of the external observer, through resin infiltration [15]. In fact, when the microporosities are filled with resin infiltrant (RI 1.46) the difference in the RI between the porosities appears the same as the surrounding healthy enamel; the hypomineralized enamel has an RI between 1.00 and 1.33, while following resinous infiltration it acquires an RI equal to 1.52, with a minimal difference compared to healthy enamel (RI 1.63) which is not perceptible to the human eye [15]. In this report, a significant improvement was achieved in the appearance and color uniformity of the treated elements in cases of mild or moderate fluorosis (Figure 2) and in cases of mild MIH (Figure 1 and Figure 3). The difficulty in managing these lesions is mainly related to the topographical location of the defect within the enamel. The resin infiltrant is a photopolymerizable material with very low viscosity, low contact angles on the enamel, high surface tensions [16], and the ability to rapidly penetrate into the porosity of the enamel. However, success depends on the thickness of the mineralized surface layer in preventing opacities in the enamel attributable to MIH, which usually extend deep inside the enamel, can be localized in the internal parts of the enamel, and can expand throughout its thickness [3,6], making the volume of the defect not very permeable [17]. For these reasons, in the present study a “clinically unsatisfactory” result was recorded in cases of moderate MIH. On the other hand, in cases of post-traumatic lesions, the “clinically unsatisfactory” result (Figure 4) is probably attributable to the acute angle that is created in this type of lesion with the superficial layer of enamel, for which infiltration with resin fluid is often incomplete at the margins, creating a visual contour of the lesion known as the “margin effect” [3,18]. In these cases, it might be useful to associate this method with microabrasion techniques [19] and/or dental bleaching [20] before resin infiltration. It is interesting to note that in cases where a “clinically sufficient” result was not found (Figure 4B), an improvement was nevertheless detected at the follow-up, probably due to the continuous infiltration over time of the fluid resin inside of the enamel structure (Figure 4C,D). In addition, stability of the results obtained at the two-year follow-up was found, agreeing with the results of an in vivo study with a six-year follow-up [21] and an in vitro study [22] in which the elements treated with infiltrative technique, after being subjected to pH changes or to common home oral hygiene techniques, showed better color stability than control cases [22]. However, color variations were found following exposure to red tea or black coffee [23]. Moreover, the high porosity of the hypomineralized enamel allows gram+ bacteria to penetrate inside the dentinal tubules, causing an inflammatory response of the pulp organ; this can cause significant hypersensitivity [24,25], sometimes hindering even simple home oral hygiene practices. In this regard, in agreement with Nogueira et al. [26], the results of the present study show a significant improvement in dentinal hypersensitivity and in the mechanical characteristics of the enamel of the elements treated by the resin infiltration technique [8,27,28]. In fact, according to in vitro studies, the ability of the second application of infiltrating resin has been demonstrated to increase the microhardness values by compensating for the polymerization contraction, thus favoring greater filling of the microporosities [8]. However, the aesthetic results of the resin infiltration technique cannot be accurately predicted due to the lack of tools and techniques suitable for carrying out a careful, precise, and predictable preoperative assessment of the depth of enamel opacities, which is fundamental to the choice of therapeutic plan. Technically, the operator seems to have control only over the topographical characteristic of the lesion, this being the only modifiable factor. The transillumination technique [29] can help the operator to distinguish the deeper areas of the lesion from the more superficial without providing precise information. The superficial infiltration technique, although not the only procedure considered for treatment of enamel opacities [14,30,31,32,33], currently represents a very satisfactory and conservative technique from both an aesthetic point of view and as a function of the shorter working time, stability of the result, minor invasiveness, reduction of hypersensitivity, and improvement of structural resistance [6,8,9,15,16,18,19,20,21].

## 5. Conclusions

The superficial infiltration technique can be considered a valid alternative to traditional techniques, especially for the treatment of enamel opacities attributable to mild or moderate fluorosis and mild MIH, obtaining optimal aesthetic results in just one session. The limit of this technique is the lack of predictability with respect to the aesthetic results. Further studies are needed in order to improve this technique and achieve a predictable and effective diagnostic–therapeutic approach in all types of enamel opacity.

## Figures and Tables

**Table 1:** Descriptive statistics. Absolute frequencies and percentages are for total sample if not otherwise indicated.

Variable	N (%)
DDE Type	
1. Moderate or low-grade MIH	24 (72.7)
2. Moderate or low-grade Fluorosis	5 (15.2)
3. Trauma	4 (12.1)
Treated teeth (mean; 95% CI)	3.5 (2.9–4.0)
Age in years (mean; 95% CI)	11 (10.4–11.6)

**Table 2:** Descriptive statistics. Absolute frequencies and percentages for total sample of repeated measurements over time of FDI, Wong–Baker Faces Pain Rating Scale, and Shiff air index.

Index/Variable			Values			
FDI	1	2	3	4	5	
Time 0			6 (18.2)	16 (48.5)	11 (33.3)	
Time 1	20 (60.6)	6 (18.2)	2 (6.1)	5 (15.1)		
Time 2	20 (60.6)	7 (21.2)	4 (12.1)	2 (6.1)		
Wong Scale	0	2	4	6	8	10
Time 0	14 (42.4)	7 (21.2)	4 (12.1)	1 (3.1)	5 (15.1)	2 (6.1)
Time 1	26 (78.8)	4 (12.1)	3 (9.1)			
Time 2	28 (84.9)	5 (15.1)				
Shiff Air Index	0	1	2	3		
Time 0	17 (51.5)	5 (15.1)	3 (9.1)	8 (24.3)		
Time 1	27 (81.8)	4 (12.1)	2 (6.1)			
Time 2	27 (81.8)	6 (18.2)				

**Table 3:** Mixed model regression on FDI.

Variable	Coefficient	95% C.I.	*p*-Value
DDE Type			
1. Moderate or low-grade MIH	Reference		
2. Moderate or low-grade Fluorosis	0.6	(−0.1, −1.3)	0.09
3. Trauma	0.5	(−0.3, 1.3)	0.2
Time			
0	Reference		
1	−2.5	(−2.8, −2.2)	<0.001
2	−2.5	(−2.8, −2.3)	<0.001
Interaction WSL Type × Time			
1 × 0	Reference		
2 × 1	−0.9	(−1.6, −0.2)	0.009
2 × 2	−0.9	(–1.5, −0.2)	0.012
3 × 1	2	(1.3, 2.7)	<0.001
3 × 2	1.3	(0.6, 2.0)	0.001

**Table 4:** Mixed model regression on Wong–Baker Faces Pain Rating Scale.

Variable	Coefficient	95% C.I.	*p*-Value
DDE Type			
1. Moderate or low-grade MIH	Reference		
2. Moderate or low-grade Fluorosis	−0.5	(−2.5, 1.6)	0.7
3. Trauma	−2.3	(−4.5, 0.1)	0.052
Time			
0	Reference		
1	−2.4	(−3.3, −1.5)	<0.001
2	−2.8	(−3.7, −1.9)	<0.001
Interaction WSL Type × Time			
1 × 0	Reference		
2 × 1	−0.4	(−2.6, 1.8)	0.7
2 × 2	0.1	(−2.2, 2.2)	0.9
3 × 1	1.4	(−1.0, 3.8)	0.3
3 × 2	1.8	(−0.6, 4.2)	0.1

**Table 5:** Mixed model regression on Shiff Air Index.

Variable	Coefficient	95% C.I.	*p*-Value
DDE Type			
1. Moderate or low-grade MIH	Reference		
2. Moderate or low-grade Fluorosis	0.3	(−0.5, 1.1)	0.5
3. Trauma	−0.6	(−1.5, 0.3)	0.2
Time			
0	Reference		
1	−0.8	(−1.2, −0.5)	<0.001
2	−0.9	(−1.2, −0.5)	<0.001
Interaction WSL Type × Time			
1 × 0	Reference		
2 × 1	−0.2	(−1.0, 0.7)	0.7
2 × 2	−0.3	(−1.2, 0.5)	0.4
3 × 1	0.3	(−0.6, 1.3)	0.5
3 × 2	0.4	(−0.5, 1.3)	0.4

## Data Availability

Database is available on request from the corresponding author.
